# Trends in late HIV diagnosis among men who have sex with men in Jiangsu province, China: Results from four consecutive community-based surveys, 2011-2014

**DOI:** 10.1371/journal.pone.0172664

**Published:** 2017-03-09

**Authors:** Haiyang Hu, Hongjing Yan, Xiaoyan Liu, Xiaoqin Xu, Jinshui Xu, Tao Qiu, Ling-en Shi, Gengfeng Fu, Xiping Huan, Willi McFarland, Chongyi Wei

**Affiliations:** 1 Institute of HIV/AIDS/STI Prevention and Control, Jiangsu Provincial Center for Diseases Control and Prevention, Nanjing, China; 2 Department of Epidemiology and Biostatistics, and Global Health Sciences, University of California, San Francisco, California, United States of America; 3 San Francisco Department of Public Health, San Francisco, California, United States of America; Centers for Disease Control and Prevention, UNITED STATES

## Abstract

**Objectives:**

To examine trends in HIV testing, late HIV diagnosis and associated factors among men who have sex with men (MSM) in Jiangsu province, China.

**Methods:**

Four consecutive community-based cross-sectional surveys were conducted among MSM from 2011 to 2014 in eight cities in the province. Participants were recruited from MSM venues and via the internet. HIV bio-behavioral surveys were conducted to collect demographic and behavioral data and measure HIV infection. HIV-infected participants with CD4 counts less than 350 cells/uL were defined as having a late HIV diagnosis. Chi-square trend tests were used to compare temporal changes over the years and multivariable logistic regression analyses were used to identify factors associated with late diagnosis.

**Results:**

A total of 2,441, 2,677, 2,591 and 2,610 participants were enrolled in 2011, 2012, 2013 and 2014, respectively. Testing for HIV in the last 12 months decreased over the time period, from 59.9% to 52.5% (*p*<0.001). Late HIV diagnosis remained high and steady, ranging from 33.3% to 44.2% over the years with no significant change over time (*p* = 0.418). MSM who were older than 24 years (aOR = 1.748, *p* = 0.020 for 25–39 years old; aOR = 3.148, *p*<0.001 for 40 years old or older), were recruited via internet (aOR = 1.596, *p* = 0.024), and did not have an HIV test in the past 12 months (aOR = 3.385, *p*<0.001) were more likely to be late diagnosed.

**Conclusions:**

Our study showed a plateau in HIV testing among MSM in China, in parallel to high levels of late diagnosis. Emerging and innovative strategies such as HIV self-testing and reaching more MSM by internet, both highly acceptable to MSM in China, may reduce late diagnosis.

## Introduction

The HIV epidemic in China is now concentrated among men who have sex with men (MSM), who account for a third and rising proportion of new HIV infections [1]. Data from national sentinel surveillance sites showed that overall HIV prevalence among MSM has been increasing steadily, from 5.7% in 2010 to 7.5% in 2013 [2,3]. Jiangsu province, located in the southeastern part of China, reported that a total of 15,039 HIV positive cases had been diagnosed by the end of September 2014. Similar to the national trend, increasing numbers of HIV-positive MSM have been detected throughout the province in the past decade. High HIV prevalence (16.0% and 13.6%) and incidence (13.59 and 12.62 per 100 person-years) among MSM in two major provincial cities have also been reported [4]. Although some studies reported that rates of condomless anal sex among Chinese MSM have been declining [5,6,7,], it is clear that promoting condom use alone is insufficient to effectively curb the HIV epidemic among Chinese MSM and other innovative and effective HIV prevention strategies should be developed and implemented.

Recent initiatives have focused attention on HIV prevention measures that expand HIV testing uptake in key populations, detect HIV cases early, and link HIV-infected individuals to care and more timely antiretroviral therapy (ART) in order to prevent onward transmission and decrease incidence at the population level (i.e., treatment as prevention) [8,9]. However, two meta-analyses showed that HIV testing rates in the past 12 months ranged from only 38% to 43.7% in Chinese MSM [10,11]. While the trend has generally been increasing, testing remains far below rates reported from many developed countries with stabilized HIV epidemics among MSM, such as 60–70% in Australia, 73% in France, and 89% in the United States [12]. With low HIV testing uptake, late HIV diagnosis or late presentation (“used to refer to all HIV-infected people who enter care at a stage of their disease where current guidelines suggest that they are unable to fully benefit from antiretroviral treatment” [13]) may be a significant barrier to the treatment as prevention approach among MSM in China. A recent national study showed that 58.8% of newly diagnosed HIV patients from 2006 to 2012 had a baseline CD4 cell counts less than 350 cells/uL [14]. Another study conducted in a city of southwestern China reported that 72.6% of newly diagnosed cases had a baseline CD4 cell counts less than 200 cells/uL [15].

However, data on late diagnosis among Chinese MSM is limited particularly at the population or community level. High levels of late HIV diagnosis, where HIV-infected MSM may unknowingly transmitting the virus to their sex partners, could be one of the main drivers of the observed high HIV prevalence and incidence among Chinese MSM. Therefore, detecting late diagnosed cases could have significant implications at the population level (i.e., reducing HIV incidence). In this paper, we analyzed data collected from four consecutive community-based HIV bio-behavioral surveys to examine trends in late HIV diagnosis and to identify associated factors among MSM in Jiangsu province.

## Methods

### Study design and participants

As a part of the routine national HIV sentinel surveillance program, a cross-sectional survey was conducted among MSM in Jiangsu province annually between April and July from 2011 to 2014. Municipal Centers for Disease Control and Prevention (CDCs) were responsible for participant recruitment, data and specimen collection, HIV screening tests, and data entry. MSM were enrolled from eight sentinel surveillance sites located in eight cities in the province. Eligible participants were biologically male, 18 years old or older, and reported anal or oral sex with another male within the past year. Participants completed a structured interview and blood specimen collection.

### Sampling and recruitment

Two convenience sampling methods were used to recruit MSM participants at the sentinel surveillance sites. The first method approached MSM at venues. Staff from the local CDCs went to MSM venues to conduct on-site survey. Owners of the venues (e.g., bars, bathhouses, and clubs) and MSM volunteers who were knowledgeable of sex-on-premise venues (e.g., parks or public bathrooms) referred interested participants to the recruiters/interviewers. Questionnaires and blood draws were conducted at these venues. The second method used internet-based sampling. Participants were recruited by local CDC staff in partnership with local community-based organizations (CBOs) via internet-based recruitment. Information including eligibility, testing sites, survey period and contact information for inquiry was disseminated through the most well-known local gay websites and most popular QQ chat room groups (QQ is the most widely used chat room/messenger software in China) to invite MSM for participation. Eligible participants came to a local CDC office to complete the questionnaires and blood draw.

All participants provided signed informed consent. Participants’ cell phone numbers and/or QQ numbers (email addresses) were obtained for HIV results notification and referrals to HIV-related resources and services. In order to prevent duplicate participation, same interviewers were staffed at each study site to identify participants (e.g., face, special characteristics). In addition, staff asked participants if they already participated in similar surveys, and used computers to cross-check participants’ cell phone numbers and/or QQ numbers for duplication before the interviews. In our study, there were no duplicate participation between venue- and Internet-based recruitment.

### Data collection

Participants were interviewed face-to-face by interviewers who completed provincial surveillance training each year. The questionnaire included socio-demographic information (e.g., age, marital status, registered residence, educational level), HIV-related behaviors such as condomless anal sex with male partners in the past 6 months, HIV knowledge questions, HIV testing in the past 12 months, and history of sexually transmitted infections (STIs) in the past 12 months. In this analysis, condomless anal sex was defined as failure to consistently use condoms with male partners in the past 6 months. Eight questions assessed participants’ HIV knowledge, where comprehensive knowledge was defined as correctly answering 6 or more questions [16].

### Laboratory methods

A volume of 3–5 ml venous blood was collected from each participant for HIV testing regardless of their self-reported HIV status per guidelines of the sentinel surveillance program. Plasma HIV antibody was first tested by an enzyme-linked immunosorbent assay (ELISA; Zhuhai Livzon Diagnostics Inc., China) which was highly sensitive in order to prevent false negatives. All initially screened HIV positive samples were retested by another ELISA reagent (Beijing Wantai Biological Pharmacy Enterprise Co., Ltd., China) which was highly specific in order to decrease chance of false positives and to reduce costs of confirmatory HIV testing. These were standard lab procedures required by the National Center for HIV/AIDS Prevention and Control for the sentinel surveillance program. If both tests were positive, participants were then contacted by CDC staff to draw a second blood sample for confirmatory testing (WB, western blot assay, MP Biomedical Asia Pacific Pte. Ltd., Singapore). For those who could not be contacted for the second blood draw, their first blood draw samples were used for confirmatory testing. We defined HIV infection as having a positive result from the Western Blot confirmatory testing. For those who could be contacted, their national unique identity numbers (ID numbers) and related information such as name and home address were collected for case checking (previously diagnosed versus newly diagnosed) before reporting to the national web-based HIV/AIDS case-reporting and management system [17]. For individuals who were confirmed HIV positive but without records in the case-reporting system, they were reported as newly diagnosed cases. Local CDCs subsequently conducted CD4 testing and follow-up care to these new patients within six months of diagnosis according to national guidelines and entered their CD4 count data into the case-reporting system. In this study, late HIV diagnosis was defined as having a baseline CD4 cell count less than 350 cells/uL within six months of diagnosis [13].

### Statistical analysis

Questionnaires at each sentinel surveillance site were double-entered and checked for accuracy using Epi Data software (version 3.1, Epi Data Association, Odense, Denmark). Chi square tests and Chi square trend tests were used to compare differences between years and observe trends over time. Factors associated with late HIV diagnosis were first assessed using bivariate logistic regression analysis. Variables with *p* values< 0.20 were entered into multivariable logistic regression models. Multivariable analysis was conducted using forward method in order to determine adjusted odds ratios (aORs). *P* values< 0.05 were considered as a statistically significant. All analyses were conducted using SPSS software (version 19.0, SPSS Inc., Chicago, IL, USA).

### Ethical approval

Analysis of the national HIV sentinel surveillance survey was approved by Institutional Review Board of National Center for AIDS/STD Control and Prevention, Chinese Center for Disease Control and Prevention (#X140121318). Authors of this study were not directly involved in obtaining blood samples or conducting interviews with participants. Data from the case-reporting and management system were de-identified and the analysis was exempt from IRB approval.

## Results

### Study population

**Fig 1:**
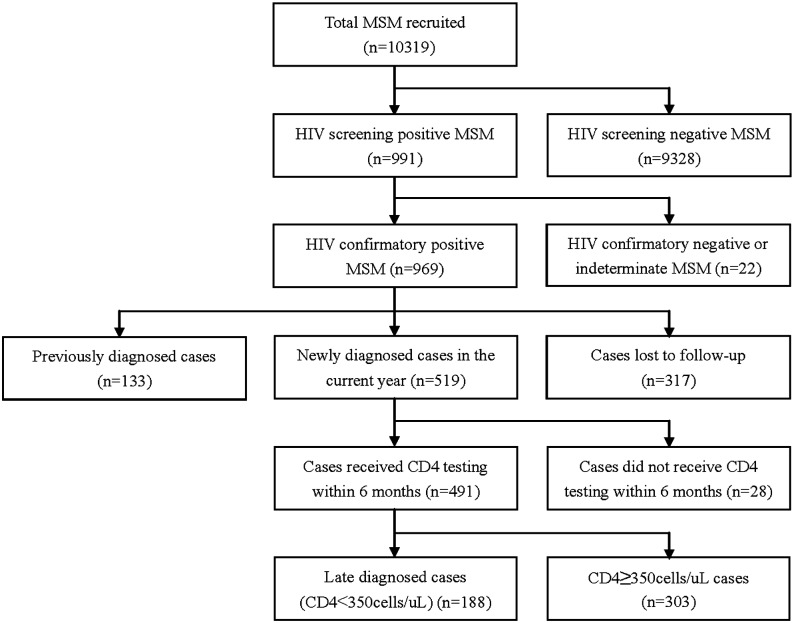
Study flowchart of MSM in sentinel surveillance surveys in Jiangsu province, China, 2011–2014.

Overall, 10,319 eligible MSM (2,441, 2,677, 2,591 and 2,610 in 2011, 2012, 2013, 2014, respectively) were enrolled into the study (Fig 1). Over all years, 991 participants were screened HIV positive, among whom 969 were confirmed HIV positive, 21 tested indeterminate, and 1 tested negative by Western Blot. Of the 969 confirmed positive MSM, 317 (32.7%) were lost to follow-up, 133 (13.7%) were previously diagnosed cases, leaving 519 (53.6%) newly diagnosed cases. Of the newly diagnosed cases, 491 (94.6%) had a baseline CD4 count measured within 6 months of diagnosis. Ofthese, 188 (38.3%) had a baseline CD4 count less than 350 cells/uL.

### Trends in socio-demographic characteristics and HIV-related behaviors

Over the four year period, increases were noted in the proportion of MSM who were single, divorced or widowed (*p* = 0.040), were not registered Jiangsu residents (*p*<0.001), had an educational level of college or higher (*p*<0.001), and were recruited via the internet (*p*<0.001) (Table 1). Condomless anal sex in the past six months decreased slightly from 44.7% in 2011 to 41.3% in 2014 (*p* = 0.036). Rates of HIV testing in the past 12 months declined from 59.9% in 2011 to 52.5% in 2014 (*p* = 0.001).

**Table 1:** Trends in socio-demographic characteristics and HIV-related behaviors of MSM in sentinel surveillance surveys in Jiangsu province, China, 2011–2014.

Variable	2011 (*N* = 2,441) *N* (%)	2012 (*N* = 2,677) *N* (%)	2013 (*N* = 2,591) *N* (%)	2014 (*N* = 2,610) *N* (%)	Trend test, *P*-value
**Age group (years)**					0.075
18–24	891 (36.5)	859 (32.1)	918 (35.4)	926 (35.5)	
25–39	1140 (46.7)	1274 (47.6)	1233 (47.6)	1111 (42.6)	
≥ 40	410 (16.8)	544 (20.3)	440 (17.0)	573 (22.0)	
Marital status*					0.040
Single, divorced, widow	1644 (67.3)	1708 (63.8)	1846 (71.2)	1765 (67.6)	
Married or cohabiting	797 (32.7)	969 (36.2)	745 (28.8)	845 (32.4)	
**Registered residence**					<0.001
Jiangsu province	1872 (76.7)	2132 (79.6)	1953 (75.4)	1907 (73.1)	
Other province	569 (23.3)	545 (20.4)	638 (24.6)	703 (26.9)	
Education level*					<0.001
Junior high or less	545 (22.3)	623 (23.4)	534 (20.6)	527 (20.2)	
Senior high school	990 (40.6)	969 (36.3)	1019 (39.3)	956 (36.6)	
College or higher	906 (37.1)	1076 (40.3)	1038 (40.1)	1127 (43.2)	
Recruitment source*					<0.001
Venues	1461 (59.9)	1804 (67.4)	1363 (52.6)	1312 (50.3)	
Internet	980 (40.1)	873 (32.6)	1228 (47.4)	1298 (49.7)	
**Comprehensive HIV knowledge**	2257 (92.5)	2531 (94.5)	2410 (93.0)	2395 (91.8)	0.088
Condomless anal sex, past 6 months*	1084 (44.7)	1073 (40.3)	1062 (41.0)	1075 (41.3)	0.036
STI, past 12 months*	161 (6.6)	119 (4.5)	93 (3.6)	153 (5.9)	0.136
**HIV testing, past 12 months**	1463 (59.9)	1240 (46.3)	1364 (52.6)	1371 (52.5)	0.001

Note:

*Data missing or refusal;

STI, sexually transmitted infections.

### Trends late HIV diagnosis among newly diagnosed MSM

A total of 206 (8.4%), 258 (9.6%), 228 (8.8%), and 277 (10.6%) participants were confirmed HIV positive in 2011, 2012, 2013, and 2014, respectively, showing a significant increase (*p* = 0.029) (Table 2). Excluding previously diagnosed MSM and those who were lost to follow-up, there were 85, 134, 126 and 174 newly diagnosed cases in each survey year, respectively, a significant increasing trend (*p*<0.001) (Table 2). There were significantly higher proportions of MSM receiving CD4 testing within six months over the years, from 36.4% in 2011 to 60.6% in 2014 (*p*<0.001). The proportion of late HIV diagnosis ranged from 33.3% in 2011 to 44.2% in 2013 and to 38.1% in 2014, with no significant temporal trend over the years (*p* = 0.418).

**Table 2:** Trends in HIV prevalence and late diagnosis among MSM in sentinel surveillance surveys in Jiangsu province, China, 2011–2014.

Variable	2011 *N* (%)	2012 *N* (%)	2013 *N* (%)	2014 *N* (%)	*P* value*
**HIV prevalence**	206 (8.4)	258 (9.6)	228 (8.8)	277 (10.6)	0.029
**Previously diagnosed cases**	49 (23.8)	26 (10.1)	30 (13.2)	28 (10.1)	<0.001
**Cases lost to follow-up**	72 (35.0)	98 (38.0)	72 (31.6)	75 (27.1)	0.018
**Newly diagnosed cases in the current year**	85 (41.3)	134 (51.9)	126 (55.3)	174 (62.8)	<0.001
**Cases who received CD4 testing within 6 months**	75 (36.4)	128 (49.6)	120 (52.6)	168 (60.6)	<0.001
**Late diagnosed cases (CD4<350 cells/uL)**	25 (33.3)	46 (35.9)	53 (44.2)	64 (38.1)	0.418

*Chi-square test for linear trend.

### Factors associated with late HIV diagnosis

In bivariate analysis, late diagnosis was significantly associated with older age, married or cohabiting, internet recruitment, and not testing for HIV in the past 12 months (Table 3). In the multivariate analysis, MSM who were older than 24 years of age (aOR = 1.75, 95% CI: 1.09–2.80, *p* = 0.020 for 25–39 years; aOR = 3.15, 95% CI: 1.81–5.47, *p*<0.001 for 40 and older), were recruited via the internet (aOR = 1.60, 95% CI: 1.06–2.40, *p* = 0.024), and did not have an HIV test in the past 12 months (aOR = 3.39, 95% CI: 2.11–5.42, *p*<0.001) were more likely to be diagnosed late.

**Table 3:** Factors associated with late diagnosis among HIV-infected MSM in sentinel surveillance surveys in Jiangsu province, China, 2011–2014.

Variable	CD4≥350 *N* (%)	CD4<350 *N* (%)	OR (95% CI)	aOR (95% CI)
**Age group (years)**				
18–24	108 (35.6)	40 (21.3)	1	1
25–39	143 (47.2)	89 (47.3)	1.680 (1.073–2.633)	1.748 (1.093–2.796)
≥ 40	52 (17.2)	59 (32.4)	3.063 (1.821–5.154)	3.148 (1.812–5.470)
**Marital status**				
Single, divorced or widowed	241 (79.5)	134 (71.3)	1	
Married or cohabiting	62 (20.5)	54 (28.7)	1.566 (1.028–2.388)	
**Registered residence**				
Jiangsu province	210 (69.3)	143 (76.1)	1.407 (0.930–2.130)	
Other province	93 (30.7)	45 (23.9)	1	
**Education level**				
Junior high school or lower	89 (29.4)	51 (27.1)	1	
Senior high school	101 (33.3)	56 (29.8)	0.968 (0.602–1.555)	
College or higher	113 (37.3)	81 (43.1)	1.251 (0.800–1.956)	
**Recruitment source of participants**				
Venues	140 (46.2)	66 (35.1)	1	1
Internet	163 (53.8)	122 (64.9)	1.588 (1.091–2.310)	1.596 1.063–2.396)
**Comprehensive HIV knowledge**				
No	24 (7.9)	9 (4.8)	1	
Yes	279 (92.1)	179 (95.2)	1.711 (0.777–3.765)	
**Condomless anal sex in the past 6 months**				
No	98 (32.3)	75 (39.9)	1.388 (0.951–2.027)	
Yes	205 (67.7)	113 (60.1)	1	
**STI in the past 12 months**				
No	273 (90.4)	163 (86.7)	1	
Yes	29 (9.6)	25 (13.3)	1.444 (0.817–2.551)	
**HIV testing in the past 12 months**				
No	181 (59.7)	159 (84.6)	3.696 (2.339–5.840)	3.385 (2.114–5.421)
Yes	122 (40.3)	29 (15.4)	1	1

## Discussion

Late diagnosis of HIV remained stable but high for MSM in Jiangsu province, China, from 2011 to 2015. Within our data, the finding is consistent with plateauing rates of HIV testing in the past year during the same period and that late diagnosis was significantly associated with lack of recent testing. Given the priority of the Chinese government’s response to the HIV epidemic among MSM (e.g., expanding HIV testing, increasing coverage of care and treatment) [18], our observed plateau in HIV testing and resulting high rate of late diagnosis indicate that current HIV testing programs have not been effectively reaching certain segments of the MSM populations that need to be tested or tested more frequently.

Another possible explanation for observing high and steady rates of late diagnosis could be that in our study, participants in later years were recruited through the internet, which was significantly associated with late diagnosis. The internet may reach MSM who do not access fixed or outreach HIV testing programs. However, the thrust of intervention programs have been mainly through outreach to MSM at venues by community-based organizations [18]. MSM who do not frequent these venues but are online may not have been reached by these prevention activities for testing, diagnosis, and linkage to care. Several other studies have reported that Chinese MSM recruited online were less likely to have been recently tested for HIV compared to those recruited at MSM venues [19,20]. Our data also showed that the number of new cases increased over the period as more and more MSM were recruited via the internet. These findings suggest that continued and enhanced efforts should be made to mobilize online MSM to get tested for HIV.

Several limitations of this study should be noted. First, about one-third of HIV-positive participants did not return for confirmatory testing, which may bias estimates of late diagnosis. However, participants lost to follow-up were not significantly different from those who were retained in terms of key demographic and behavioral characteristics. Second, our definition of late diagnosis used a time period of 6 months instead of shorter time periods. A recent study suggested that a one-month time period would be more reliable [21]. Third, participants might have provided socially desirable answers to sensitive questions due to face-to-face interviewer administered survey mode which could have underestimate certain risk behaviors. To minimize this bias, interviewers from all MSM sentinel surveillance sites were trained annually and followed strict interviewing protocol. Finally, our results may not be generalizable to all MSM in the province and other regions due to the convenience sampling methods and hence selection bias.

In conclusion, our data demonstrated that HIV testing promotion should continue to be a top priority on the HIV prevention agenda for MSM in China. If newly emerging trends of complacency in testing and continuing high levels of late diagnoses prove true, our data should serve as a call for innovative ways to reach MSM who have never tested and to facilitate frequent testing among MSM at risk. Emerging and innovative strategies such as HIV self-testing and reaching more MSM by the internet, which are highly acceptable among Chinese MSM [22,23], should be implemented to increase HIV testing coverage and early detection, reduce proportion of late diagnosis, improve patient outcomes and reduce HIV transmission. Previous studies also showed that social discrimination, fear of testing HIV positive, and lack of testing resources are major barriers to accessing HIV testing [24,25]. Intervention programs, such as health education campaigns and improved training of HIV testing counselors, should take into consideration these factors in order to create a friendly and supportive environment to encourage HIV testing among MSM communities.

## Supporting information

S1 File MSM sentinel surveillance questionnaire (English). (DOCX)

S2 File IRB approval letter. (PDF)

S3 File Data. (XLSX)
