# Supplementary material for: Trends in late HIV diagnosis among men who have sex with men in Jiangsu province, China: Results from four consecutive community-based surveys, 2011-2014
Source: PLoS One. 2017 Mar 9;12(3):e0172664. doi: 10.1371/journal.pone.0172664 (PMC5344382; doi:10.1371/journal.pone.0172664)
Supplement: S1 File — (DOCX) [file pone.0172664.s001.docx]

### MSM Questionnaire

Questionnaire on Health

A01 Survey site province city county

A02 District code □□□□□□

A03 Questionnaire number □□□（001—999）

A04 Date of survey □□□□ year□□ month□□ day

A05 Recruitment source ①bar/disco/club ②bathhouse ③park/public bathroom ④internet ⑤other( please specify)

Hi, my name is……, from……. We are conducting a survey. The purpose is to understand people’s knowledge and behavior related to health. Please be relax, this survey is anonymous, we will keep your answer secret, so hope you answer them based on your real situation. It will take you 10 minutes to answer these questions. I can provide counseling for you when finish the interview if you need (for example, you can ask some questions related to health). Hope you support our work. Thank you very much!

Ask participants: Have you ever participated in this survey recently? If answered “yes”, stop the investigation.

B01 Year of birth year

B02 Marital status with female ①single ②married ③cohabiting ④divorced or widow

B03 Registered residence ①Jiangsu province ②Other province( please specify) ③ Foreigners(please specify country, Skip to B05)

B04 Nationality

B05 Time living in local area: ①＜3 months ②3~6months  ③7~12months ④1 ~2years ⑤＞2years

B06 Education level ①illiteracy ②primary school ③Junior high school ④Senior high school ⑤College or higher

C01 Can a person infected with HIV be observed from outside? ①can ②cannot③don’t know

C02 Can mosquito bites spread HIV/AIDS? ①can ②cannot ③don’t know

C03 Can eating together with HIV/AIDS patients be infected with HIV? ①can ②cannot ③don’t know

C04 Can person be infected with HIV through blood transfusion containing HIV virus? ①can ②cannot ③don’t know

C05 Can sharing syringes with HIV/AIDS patients be infected with HIV? ①can ②cannot ③don’t know

C06 Can baby be infected with HIV through HIV positive woman to child route? ①can ②cannot ③don’t know

C07Can using condoms correctly reduce spread of HIV/AIDS? ①can ②cannot ③don’t know

C08 Can having sex with only one partner reduce spread of HIV/AIDS? ①can ②cannot ③don’t know

D01 In the past 6 months, did you have anal sex with male partners? ①yes ②no (skip to F01) ③refusal

D02 In the last week, how many times did you have anal sex with male partners? ① times ②refusal

D03 In the past 6 months, did you use condoms during the last anal sex with male partners? ①yes ②no ③refusal

D04 In the past 6 months, how often did you use condoms during having anal sex with male partners? ①never②sometimes ③always ④refusal

E01 In the past 6 months, did you have commercial sex with male partners? ①yes ②no ( skip to F01) ③refusal

E02 In the past 6 months, how often did you use condoms during having commercial sex with male partners? ①never ②sometimes ③always ④refusal

E03 In the past 6 months, did you use condoms during the last commercial anal sex with male partners? ①yes ②no ③refusal

F01 In the past 6 months, did you have sex with female partners? ①yes ②no (skip to G01) ③refusal

F02 In the past 6 months, how often did you use condoms during having sex with female partners? ①never ②sometimes ③always ④refusal

F03 In the past 6 months, did you use condoms during the last intercourse with female partners? ①yes ②no ③refusal

G01 Did you take drugs? ①yes ②no (skip to H01) ③refusal

G02 Did you inject drugs? ①yes ②no ((skip to H01) ③refusal

G03 Did you share syringes with others? ①yes ②no (go to H01) ③refusal

G04 In the past 6 months, how often did you share syringes with others? ①never ②sometimes ③always ④refusal

H01 In the past 12 months, have you ever been diagnosed with a sexually transmitted diseases (STDs)? ①yes ②no (skip to I01) ③refusal

H02 In the past 12 months, which type of STDs were you diagnosed? ( multi-choice )

①gonorrhea ②syphilis ③chlamydial trachomatis ④condyloma acuminata ⑤herpes progenitalis ⑥others ( please specify) ⑦refusal

In the past 12 months, have you ever received the following HIV/AIDS prevention services?

I01 Condom promotion and provision/HIV counseling and testing ①yes ②no

I02 Methadone maintenance treatment/clean syringes exchange ①yes ②no

I03 Peer education ①yes ②no

J03 When did you test HIV in the last time? year month (if never, do not fill in, end)

J04 The result of your HIV testing in the last time ①negative ②positive ③don’t know

Interview is over, thank you for your cooperation. In order to understand your health status, we need to collect your blood to conduct HIV, syphilis and HCV test.

T01 Whether the blood is collected in the survey ①yes ②no

T02a Whether the participant previously diagnosed HIV positive ①yes ②no (skip to T03)

T02b If yes, the earliest date of the HIV confirmation: year month

T03 The result of the HIV antibody test: The first ELISA ①positive ②negative (skip to T04)

The second ELISA ①positive ②negative

HIV confirmatory test ①positive ②negative ③indeterminate ④no detection

T04 The result of syphilis test ELISA ①positive ②negative (go to T05)

RPR/TRUST ①positive ②negative

T05 The result of HCV antibody test The first ELISA ①positive ②negative (over)

The second ELISA ①positive ②negative

Investigator signature Supervisor signature
