# Supplementary material for: Trends in late HIV diagnosis among men who have sex with men in Jiangsu province, China: Results from four consecutive community-based surveys, 2011-2014
Source: PLoS One. 2017 Mar 9;12(3):e0172664. doi: 10.1371/journal.pone.0172664 (PMC5344382; doi:10.1371/journal.pone.0172664)
Supplement: S2 File — (PDF) [file pone.0172664.s002.pdf]

中国疾病预防控制中心
性病艾滋病预防控制中心

中国疾病预防控制中心
性病艾滋病预防控制中心伦理委员会
项目评审报告

项目编号：X140121318

经中国疾病预防控制中心性病艾滋病预防控制中心伦理委员会专家评审后，认为下列项目符合我国伦理有关要求，在获得数据使用授权后，项目可利用现有信息进行数据分析。

项目名称：　使用中国艾滋病监测数据开展科学研究的伦理申请

项目负责人：崔岩　研究员

单　　位：　中国疾病预防控制中心性病艾滋病预防控制中心

初审日期：　2014 年 1 月 21 日

评审日期：　2015 年 6 月 19 日

批准日期：　2015 年 6 月 19 日

本次伦理审批自 2015 年 6 月 19 日起，有效期一年。

主席：(签字)王若涛

王若涛

中国疾病预防控制中心

性病艾滋病预防控制中心伦理委员会

地址：北京市昌平区昌百路 155 号　邮编：102206　电话：58900922　传真：58900920　E-mail:office606@chinaaids.cn

中国疾病预防控制中心性病艾滋病预防控制中心
NATIONAL CENTER FOR AIDS/STD CONTROL AND PREVENTION, CHINESE CENTER FOR DISEASE CONTROL AND PREVENTION

155 Changbai Road, Changping District
Beijing 102206, P. R. China
Website: www.Chinaaids.cn

INSTITUTION REVIEW BOARD APPROVE LETTER

Project No: X140121318

PROJECT TITLE: Data Analysis of HIV/AIDS surveillance in China

PRINCIPAL INVESTIGATOR OF PROJECT: Cui Yan, Researcher

INSTITUTE: National Center for AIDS/STD Control and Prevention, Chinese Center for Disease Control and Prevention, P. R. China

FUNDING AGENCY: National Health and Family Planning Commission, Ministry of Finance of People's Republic of China

DATE FIRST SUBMITTED: Jan. 21, 2014

DATE FOR WHICH REVIEWED (THE 2ND REVIEWED): Jun. 19, 2015

DATE APPROVED: Jun. 19, 2015

The INSTITUTIONAL REVIEW BOARD of NATIONAL CENTER FOR AIDS/STD CONTROL AND PREVENTION, CHINESE CENTER FOR DISEASE CONTROL AND PREVENTION, has reviewed the proposed use of human subjects in the above-mentioned project. The right and the welfare of the subject are adequately protected; the potential risks are outweighed by potential benefits.

Our IRB, National Center for AIDS/STD Control and Prevention, Chinese Center for Disease Control and Prevention (China CDC) is registered with the U.S. Office for Human Research Protections, IRB00002276, and has a Federal wide Assurance. FWA00002958.

Signature: Ruotao Wang

Ruotao Wang

Chair, Institutional Review Board of NCAIDS, CCDC

Date: Jun. 19, 2015
